# Reflex Pains in the Trifacial Nerve

**Published:** 1909-09-15

**Authors:** Chalmers J. Lyons

**Affiliations:** Jackson, Michigan


### REFLEX PAINS IN THE TRIFACIAL NERVE.

BY CHALMERS J. LYONS, D.D.S., JACKSON, MICHIGAN.

Read before the Geo. H. Mosher Dental Club, Jackson, Mich., March 3, 19(9

The subject which I will discuss briefly to-night is "Reflex Pain in the Trifacial Nerve."

The dentist meets more cases of reflex pain in the fifth or trifacial nerve than any other practitioner of the medical art. Why? Because the field of his professional activity is almost wholly circumscribed by the distribution of this nerve. The fifth nerve is a mixed nerve but chiefly sensory to the face, teeth, eye, ear, and fore part of the scalp.

The face and scalp may be the seat of local irritation which may react reflexly as pain in the teeth and gums—likewise an irritation of the eye or external ear may cause reflex pain in any of the teeth. On the other hand, a diseased tooth may produce reflex tender points on the skin usually located where this nerve emerges through the bones or fascia or in the eye or in the ear.

The dental organs are peculiarly expoesd to infection because of the great number of teeth together with considerable mucous territory in the antrum of Highmore and Oral cavity, all of which send branching processes to the Gasserian Ganglion where the sensory fibers of the trifacial nerve spread out into a flattened somewhat fan shaped plexiform bundle. The complex communication of these nerve fibers make the dental region the point of greatest traction for reflex pain in healthy mouths as well as the point of least resistance under circumstances of local diseases in the teeth, gums or antrum. Besides the intricate system of sensory fibers of this nerve it also represents the sensory roots of all the motor cranial nerves from the third to the twelfth inclusive; hence it responds reflexly to motor nerves of the orbit face and tongue.

Pain is produced by a stronger stimulus than usual and can only be defined, and then imperfectly, by comparison with other common sensations, as hunger, thirst, or cold. Webster defines pain as an uneasy sensation in animal bodies of any degree from slight uneasiness to extreme distress or torture proceeding from pressure, tension, separation of parts by violence or any derangement of function. Pain for one is only amusement for another. One patient will almost collapse under a given pain while another with stoical indifference will take his sensations as a foregone conclusion. Many factors have got to be taken into consideration in making an estimate of what will be painful to different patients in given cases. Nationality, temperament, age, physical and mental conditions, all influence the common sensation called pain. The writer has sometimes felt that occupation had some influence over pain for he has found men in certain occupations of life could not withstand the sensation of pain as well as men in other vocations.

Pain follows nerve continuity just as blood follows continuity of vessels and is considered by Eckley as an impenderable or magnetic projectile. As a projectile, pain follows first the line of greatest traction, second the path of least resistance. This accounts for the fact that reflex pain may be felt in a tooth in a weakened condition when the cause of that pain is remote from that particular region.

The dentist of to-day should be a conservative operator the same as the educated surgeon is conservative in diagnosing and prognosing pathological conditions.

Pain in a finger does not necessarily demand amputation or even local medication, neither does pain in a tooth always demand extraction or even divitalizing of the pulp. We should first determine the cause and seat of trouble.

The pain is produced by local inflammation and is either a co-incidence of such condition or its cause, or pain is produced by local inflammation in some remote region of the body and a reflex pain results. For instance, a patient has an exposed pulp. The dentist is convinced of the cause of the pain and its location. Here is coincidence of pain and cause; or a patient may have pain in a tooth and tests show that tooth to be perfectly sound, free from any inflammation. Further examination locates the trouble in the eye or the ear or the tongue or in some other tooth, then the pain is evidently a reflex pain. Pain in any region in the absence of local inflammation is likely to be reflex and the dentist should be careful to locate positively its source and eliminate entirely all possible sources of inflammation in any given case of pain before completing his diagnosis.

In reflex pain there is always structural nerve continuity or nerve connection between the place where the patient feels the pain and the seat of irritation. Pain may be felt in a tooth when the seat of irritation may be in any of the organs supplied by the trifacial nerve or pain may be felt in one tooth when the seat of the irritation is in another tooth being supplied by a different branch of the same nerve.

An irritation in any branch of the sensory distribution of the fifth nerve may be the exciting cause of reflex pain in any other branch of the same nerve. The dentist then, must possess diagnostic skill along the anatomic lines of nerve-continuity between a reflex pain in a tooth, gum, or the antrum, and all possible places where the irritant might logically be sought.

Reflex pain in a tooth may be found not only in cases in which the irritant is in one part and the pain in another part of the fifth nerve but where there is radiation of pain from the fifth nerve to the cervical nerve and vice versa and also in which there is radiation of pain from the fifth nerve to the throacic and abdominal viscera and vice versa through the gangliated cord of the sympathetic.

As has already been stated the trifacial nerve represents the sensory roots of all the motor cranial nerves from the third to the twelfth inclusive. The connection between the cervical nerves and the trifacial possibly comes via the twelfth or hypoglossal nerve for the communicantes hypoglossi are given off from the second and third cervical nerves to form a loop with the decendens hypoglossi by a branch of the twelfth.

The radiation of pain from the thoracic and abdominal viscera via gangliated cord of the sympathetic may be accounted for from the fact that the cervical portion of the gangliated cord gives off branches to the Gasserian ganglion which gives origin to the sensory fibers of the Trifacial or fifth nerve.

The time is too limited for this paper, to consider in detail the reflex pain from the cervical nerves or from the abdominal or thoracic viscera gangliated cord of the sympathetic, suffice it to say that the irritation in these regions causing the reflex pain in a tooth may be inflammation of the skin, rheumatism in the articulation, inflammation, deep seated in muscles, bone and joint, etc. or in irritation of some remote nerve ending having communication with the trifacial.

In considering these cases in which the irritant is in one part and the reflex pain in another part, of the fifth nerve, a methodical anatomical examination should be made by first examining all the teeth on the painful side; examine the gums and alveolar process on painful side and then examine antrum of that side; also the teeth, gums, and alveolar process of opposite side as well as the opposite jaw.

The mucous membrane of lips, Stenson's duct, tongue, temporo-mandibular articulation, hard and soft palate parotid gland, all should be examined for a possible seat of irritation to this nerve before completing a diagnosis of the case.

When we take into consideration the extensive area which is influenced either directly or indirectly by the trifacial nerve, and the fact that the seat of irritation producing reflex pain in any part of the Oral Cavity may be in any of the remote nerve endings, either in this nerve or in the systems having communication with the fifth nerve, we can readily understand that the treatment of trifacial neuralgia or Tic-Douloureux is indeed a complex problem.

Under certain peculiar conditions of the system cold or damp excites an attack of neuralgia of the fifth nerve. These conditions are thought to be of a gouty or rheumatic nature and the cold seems to start a sub-acute inflammation of the sheath of the nerve; or some irritation of the peripheric organs may excite attacks of neuralgia in nervers nearly or remotely associated; for example, dental caries may be an irritant directly to the second or third division of the trifacial nerve and indirectly by reflex pain may excite supra orbital neuralgia through the first or ophthalmic devision.

Tic-douloureux or trifacial neuralgia occuring in youth and apparently as an accident of exposure or as a result of faulty teeth may never recur.

It is perhaps more common however for repetitions of the attack to take place alternating it may be with neuralgia in other quarters. It is not infrequently liable to recur, especially under circumstances of depression through a whole life time but may never have the character of extreme severity. In some cases it is not only obstinate but of terrible violence, the patient being incapacitated for many years by constantly recurring attacks. It has been known to have been violent enough to destroy life but not frequently so.

In obstinate cases of trifacial neuralgia those that can not be governed by medicinal treatment, surgical operations on the trifacial nerve is often resorted to. The operation probably most frequently made is neurectomy. This is the removal of a section of the trifacial nerve. When this fails, the branches effected may be surgically torn from both central and peripheric attachment. Sensory nerves have a peculiar function of regenerating themselves and while the operation may insure relief for a period of two or three years, permanent relief may only be effected by the removal of the Gasserian Ganglion. This operation has been occasionally performed for Tic-douloureux or neuralgia of severe type with absolute success by many of the Oral Surgeons of this country, but it is a radical operation and is attended with great risk on account of the close proximity of the internal carotid artery and the fact that this ganglion is situated in the base of the skull and many vital points have to be passed in reaching it. This is known as the Hartley Krause operation on account of these two surgeons being the first to perform it.

The principle involved in the removal of ganglia is the distruction of the ganglionic sensory cells. The sensory fibers of both spinal and cranial nerves grow inward to the cord and brain respectively from certain more or less remotely located ganglia. If the ganglion is removed the cells that report sensation are gone and the sensory life of the same is ended. In the removal of the Gasserian ganglion the sensory cells are destroyed and pain can not recur in any part of the area supplied wholly by the trifacial nerve.

By a thorough understanding of the intricate system of the trifacial nerve the dentist should be able to control to a minimal amount the pain produced on the patient

1st. He should minimize the extent of the sensory area irritated.

2nd. He should minimize the length of time that the sensory area is exposed to irritation.

These two elements physiologically figure conspicuously in the production of pain and the operator has it in his power to produce maximal or minimal pain on his patients in proportion to his observation of these physiologic precepts.

The dentist who carelessly manipulates the lips, who roughly retracts the angles of the mouth, who painfully compresses gum tissue by applying the rubber dam and who leaves the patient three hours in the chair when one hour was all that was necessary, deserves censure, for he has violated both the extent of painful area as well as the time element. These things should always be taken into consideration in performing a dental operation and if we would devote more time to the study of the anatomy and physiology of the trifacial nerve and the phenomena of reflex pain in connection thereto, we would be better diagnostitions and be more able to reduce to a minimum the amount of pain attending a dental operation, and in so doing would receive the benediction of our patients to repay us for our efforts.

